# The impact of basketball on the social adjustment of Chinese middle school students: the chain mediating role of interpersonal relationships and self-identity

**DOI:** 10.3389/fpsyg.2023.1205760

**Published:** 2023-06-28

**Authors:** Sui Haoran, Lu Tianci, Chen Hanwen, Tao Baole, Chen Yiran, Jun Yan

**Affiliations:** College of Physical Education, Yangzhou University, Yangzhou, Jiangsu, China

**Keywords:** basketball intervention, middle school students, social adjustment, interpersonal relationships, self-identity, chain mediation

## Abstract

**Background:**

This study examines the effects of 12 weeks of basketball on interpersonal relationships, self-identity and social adjustment of middle school students, as well as exploring the mediating role of interpersonal relationships and self-identity in basketball’s influence on social adjustment.

**Methods:**

A total of 87 students from a middle school in Jiangsu Province, China, were selected to participate in this study. A 12-week basketball intervention experiment was conducted, and questionnaires were administered to measure the study variables. Common method bias test, normality test, ANOVA and Pearson correlation analysis were used to analyze the study variables. The theoretical model of this study was also validated using the Process plug-in developed by Hayes, setting *p* < 0.05 (two-tail) as statistically significant.

**Results:**

After a 12-week basketball intervention experiment, the interpersonal relationships, self-identity and social adjustment of the middle school students in the experimental and control groups showed improvement, with the experimental group showing significantly more significant improvement than the control group. A 12-week basketball intervention can positively impact the social adjustment of middle school students, with interpersonal relationships and self-identity acting as a chain mediator in the impact process.

## 1. Introduction

Social adjustment is the process by which individuals’ perceptions and behaviors change in response to changes in the social environment, adapting to the social environment in which they live (Weissman, 1975). In social life, each individual has their unique way of dealing with the world and people and has aspirations and needs for interpersonal interaction, cooperation, friendship, respect, reputation and achievement, all of which depend on the individual’s social adaptation for their fulfilment. Social adjustment is an essential competency for the student population, and the development of social adjustment is an important responsibility of schools. In contrast, middle school is critical in developing students’ physical and mental health. Physical maturity creates a sense of adulthood in middle school students, who want to acquire some of the powers of the majority, find new standards of behavior and desire to change social roles. However, their minds are still immature, many expectations cannot be met, leading to mental health problems such as frustration (Yao, 2002). The study showed that while middle school students’ levels of positive adjustment were relatively stable, their negative adjustment tended to increase with grade level (Li et al., 2017). If a person is not in tune with society, they will develop a state of mind incompatible with everything in their environment, leading to psychological distortions over time. Strong social adjustment skills can help middle school students reduce stress, regulate negative emotions and control aggressive behaviors, effectively promoting mental health (Heath et al., 2017).

Scholars have widely discussed the question of how to improve young people’s social adjustment skills. A recent cross-sectional survey with a large sample showed a strong positive correlation between physical activity and social adjustment (Chen et al., 2022). Social adjustment theory and integrated adaptive development theory suggest that an individual’s adjustment skills are developed through continuous learning and exploration in life practice (Bristol, 1915). At that level, physical activity as a positive social interaction activity may improve individuals’ social adjustment. Therefore, this study also takes this as a starting point to explore the effects of physical exercise on the social adjustment of middle school students and the mechanisms of its influence using an experimental approach of basketball intervention.

There may be an essential mediating role in physical exercise to improve the social adjustment of individuals. Interpersonal relationships are the mutual psychological relationships that people establish with each other during ordinary activities to satisfy their various needs (Hartup, 1989). Social connection theory suggests that healthy adolescent development requires good connections with those around them. When relationships with others are faulty, adolescents feel less secure and regular interpersonal communication is compromised, leading to poor interpersonal and social adjustment (Hirschi, 2002). The interpersonal relationships of middle school students entering middle school have changed significantly (Fuligni et al., 2001), The interpersonal relationships of middle school students entering middle school have undergone significant changes They are less psychologically and emotionally dependent on their parents and teachers and are more eager to build good interpersonal relationships with their partners (Steinberg, 1990). Therefore, at this particular time, the interpersonal relationships of middle school students may be one of the most critical factors affecting their level of social adjustment. The theoretical model of the psychological benefits of physical exercise suggests that physical exercise can directly influence behavioral performance, such as interpersonal interactions (Tomporowski et al., 2011). Some studies have found that individuals who regularly participate in physical exercise are more likely to have improved interpersonal relationships (Kim et al., 2021), consistent with the concept proposed by this theoretical model. Thus, interpersonal relationships may mediate the pathway by which physical exercise influences middle school students’ social adjustment.

Self-Identity generally refers to “the principle orientation of an individual’s self-perception, the conviction of self-emotional experience, the monitoring of self-regulation and the fixation of self-evaluation” (Johnson, 1982). According to Erikson’s self-identity theory, the positive and stable development of self-identity is a primary psychological task during adolescence and is of great importance for adolescents’ physical and mental health and individual wellbeing (Zahn-Waxler et al., 1995). Research has shown that a positive self-identity of young people can help them adapt to society (Kim and Yang, 2019). At the same time, positive self-identity enables young people to have good personality traits, good survival skills and a healthy psychological state, to maintain a positive and enthusiastic attitude toward life to face the pressures and challenges of reality, to study hard, to work hard and to take up social responsibilities, all of which are concrete manifestations of identification with society and adaptation to culture (Gao and Feng, 2013). Research has shown that during physical exercise, individuals continue to feel positive changes in themselves and thus receive positive feedback, increasing their self-efficacy, self-confirmation and self-confidence levels (Anderson et al., 2007). This virtuous cycle increases the individual’s psychological capital, reduces low self-esteem, increases self-confidence and helps the individual develop positive and healthy values and worldviews, which in turn positively affects the individual’s self-identity.

Currently, there is a general consensus in the research on moderate intensity physical activity that moderate intensity physical activity has a positive effect on many indicators of adolescents’ mental health, physical composition and academic performance. Subramaniapillai et al. (2016). found that moderate intensity physical activity had a positive effect on adolescents with bipolar disorder. In China, Fu and Fan (2016) found that moderate-intensity physical exercise had a positive effect on the conversion and inhibition functions of executive functions in junior high school students. Qin and Yuan (2019) showed that regular moderate-intensity physical training can significantly improve the physical fitness level of university students and improve their motor skills to a certain extent, in the process, university students can constantly feel the changes in themselves, so that they can continuously obtain positive feedback, which will enhance their self-efficacy, self-affirmation and self-confidence level, and finally make university students’ inferiority complex This will lead to a reduction in low self-esteem and an increase in psychological capital. The duration of physical activity is also important for the development of individual mental health. Studies have shown that moderate-to-vigorous physical activity for ≥60 min has a positive effect on the development of psychological wellbeing (Zeng et al., 2021). In contrast, due to the dose effect, when the amount of physical exercise exceeds the threshold that an individual can tolerate, it has a negative impact on the individual’s mental health (Salmon, 2001). Accordingly, in conjunction with existing research, it has been found that adolescents who engage in 31–59 min of physical activity have better mental health than those who engage in 11–20 min of physical activity (Zhang, 2016). In terms of frequency of physical activity, Chekroud et al. (2018) analyzed the effect of time and frequency of physical activity on individuals’ physical and mental health and concluded that the positive effect of 30–60 min of physical activity three to five times per week was stronger on individuals’ mental health, whereas individuals who exercised more than 23 times per month or for more than 90 min per session had worse mental health. With regard to the periodicity of physical activity, studies have shown that the minimum period of physical activity that can produce beneficial physical and mental improvements is 10–12 weeks (Zeng et al., 2021).

**FIGURE 1:**
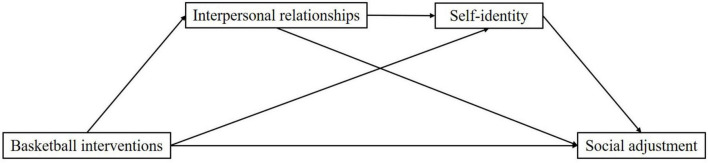
Theoretical model diagram.

In constructing the theoretical model for this study, we found that there may also be a positive relationship between interpersonal relationships and self-identity. As adolescents in middle school go through a critical period of physical and mental development, they are more likely to follow the advice of their partners rather than their parents at this stage. And numerous studies have shown that adolescents at this stage are more likely to actively seek attention and respect in their peer groups (Fuligni et al., 2001). Non-supportive peer relationships can harm academics, which can lead to psychological problems such as depression and anxiety and is detrimental to the development of self-identity (Xin et al., 2019). Therefore, interpersonal relationships may positively impact the development of an individual’s self-identity. In summary, this study uses an experimental approach of basketball intervention to explore the impact of physical exercise on the social adjustment ability of middle school students and the role and influence mechanism played by interpersonal relationships and self-identity in it, and proposes a theoretical model (Figure 1).

## 2. Materials and methods

### 2.1. Ethical approval

The study was carried out under ethical standards and was approved by the Ethical Committee of Yangzhou University Medical College (No. YXYLL-2022-123).

### 2.2. Program details

#### 2.2.1. Experimental group

Each session was organized in a variety of ways, including group work, rivalry exercises and competitions to create a complex sporting situation. The experimental group received basketball intervention 3 times a week for 12 weeks. The content of the sessions was mainly based on basic movement techniques (e.g., dribbling, moving, passing, shooting, etc.) and the exercises were completed in the form of one-to-one cooperation or multi group cooperation. After completing the basic exercises, a competition practice format is arranged to develop middle school students’ teamwork skills and their ability to apply the technical movements they have learnt to actual games. The intensity of training is determined according to the training time, interval and training volume of each group. A total of 10 min are set aside in each training session for preparation and relaxation activities.

#### 2.2.2. Control group

In the control group, the physical education teacher in charge of the class arranges the teaching content during the sports intervention experiment. The content of the arrangement is based on the school syllabus and includes gymnastics, table tennis, volleyball, quality exercises, etc. The control group’s classes were structured as a preparatory part (assembly and formation, warmup before class), a basic part (teaching content) and an end part (free activities, assembly and formation before class). The experimental participants were instructed by the same teacher at a fixed time and place, and students were asked to refrain from participating in physical activities other than those required by the school as far as possible, and parents were issued with a letter of information.

### 2.3. Sample

The minimum total sample size of 86 was calculated by estimating the sample size using G-Power 3.1.9.7 software. Therefore, a total of 92 students from two classes of the first grade of a middle school in Zhenjiang City, Jiangsu Province, were used as the subjects of this experimental study by means of convenient whole group sampling. Before the experiment, the Physical Activity Rating Scale was administered to all subjects to exclude those who had too much or too little physical activity, and the Chinese Middle School Students’ Mental Health Inventory was administered to exclude those who had problems with emotions, behavior, personality, thinking, attention and other specific mental disorders, to ensure that the physical activity and mental health levels of the subjects included in the experiment were normal. Finally, a total of 5 students with too much or too less physical activity and mental health problems were excluded. There were 87 valid questionnaires after elimination, including 42 in the experimental group and 45 in the control group, with an effective rate of 94.6%. The demographic information of the subjects in the experimental and control groups is shown in Table 1.

**TABLE 1:** Demographic characteristics.

Variables	Experimental group		Control group	
Gender	Male (*n* = 18)	Female (*n* = 24)	Male (*n* = 25)	Female (*n* = 20)
Age	13.33 ± 0.87		13.27 ± 0.84	
Height (cm)	162.43 ± 7.67		160.46 ± 7.88	
Body weight (kg)	52.17 ± 10.24		54.89 ± 16.90	
Amount of physical activity	32.64 ± 8.61		29.89 ± 8.87	

### 2.4. Research methods

A mixed experimental design 2 (group: 1 experimental group, 1 control group) × 2 (time: pre-test and post-test) was used, with the exercise intervention (experimental group) and participation in normal learning activities (control group) of middle school students as independent variables. A natural experiment with between group and within group pre-post measures for experimental and control groups, which uses social adjustment of middle school students as the dependent variable and interpersonal relationships and self-identity as mediating variables. The experimental group used the intervention of basketball, setting the exercise intensity at 60–69% of the maximum heart rate. The control group followed the requirements of the Physical Education and Health Curriculum for regular physical education classes. The same teacher instructed the experimental participants at a fixed time and place, and students were asked to refrain from participating in physical activity other than that required by the school as far as possible and were issued with an information letter.

### 2.5. Research tools

#### 2.5.1. Physical activity rating scale (PARS-3)

Using the Physical Activity Rating Scale developed by Koyo Hashimoto (Xia et al., 2018). The scale uses intensity, duration and frequency of physical activity as factors to examine the extent of physical activity participation. Level of physical activity involvement = intensity × (time −1) × frequency. Each of the three factors is divided into 5 levels and scored from 1 to 5. The maximum score for participation in sporting activities is 100, and the minimum score is 0. The level of participation in physical activity is graded as follows: small grade ≤19 points, medium grade 20 to 42 points and large grade ≥43 points. The scale has good psychometric characteristics and has been widely used in young people in Chinese culture (He et al., 2022; Zhou and Huo, 2022). The test–retest reliability in Liang (1994)study was 0.82. In this study, the Cronbach’s α coefficient for the scale was 0.83.

#### 2.5.2. Mental health scale for Chinese middle school students

Using the Mental Health Scale for Chinese middle School Students developed by Wang et al. (1997), The scale has 60 items and is scored on a Likert five-point scale, i.e., 1 for none, 2 for mild, 3 for moderate, 4 for severe and 5 for serious. The score is obtained by adding the scores of the 60 items together and dividing by 60 to give the total mean score of the subject’s mental health, which indicates the overall mental health status. If the total mean or factor score for mental health is <2, it shows a relatively healthy psychological condition; ≥2 indicates a degree of the psychological problem; and a total mean or factor score of 5 indicates a serious problem. In this study, The Cronbach’s α coefficient for the scale was 0.93, and the results of the validation factor analysis were: *X^2^/df* = 2.88, CFI = 0.93, TLI = 0.91, RMSEA = 0.05, indicating that the scale has good reliability and validity.

#### 2.5.3. Interpersonal relationship scale

Using the Interpersonal Relationship Scale developed by Wo et al. (2001), The scale has 30 questions and is scored on a Likert 5 scale. The scale has five dimensions: “peer relationships,” “opposite sex peer relationships,” “parent child relationships,” “teacher student relationships,” and “stranger relationships.” Subjects chose an appropriate rating based on their feelings over the last month. All items on the scale were negative and rated on a scale from 1 (strongly disagree) to 5 (strongly agree), and the scale was processed with reverse scoring questions before being counted. The scale’s total score is the sum of all the questions in the five dimensions, with higher scores representing higher levels of interpersonal relationships among adolescents. The Cronbach’s α coefficient for the scale was 0.88, and the results of the validation factor analysis were: *X^2^/df* = 4.70, CFI = 0.90, TLI = 0.90, RMSEA = 0.05, indicating that the scale has good reliability and validity.

#### 2.5.4. Self-identity scale

This study used the Self-Identity Scale (Ochse and Plug) developed by Oakes et al. (1986) and Burger and Chen (2014). The scale consists of 19 items on a 4-point Likert scale, 1 (not applicable) to 4 (very appropriate). The scale has been widely used by national and international researchers and has good reliability and validity. The Cronbach’s α coefficient for the scale was 0.86, and the results of the validation factor analysis were: *X^2^/df* = 5.66, CFI = 0.90, TLI = 0.90, RMSEA = 0.06, indicating that the scale has good reliability and validity.

#### 2.5.5. Social adjustment scale for adolescents and young people

Using the Social Adjustment Scale for Children and Adolescents by Guo et al. (2018), The scale consists of 35 questions and is scored using the Likert 7 scale. The scale has seven dimensions, namely “Personable,” “Autonomy in learning,” “Acceptance of views,” “Human-computer harmony,” “Group integration,” “Environmental satisfaction,” and “Independence in life.” It has been used to measure primary and middle school students with good reliability and validity. The Cronbach’s α coefficient for the scale was 0.80, and the results of the validation factor analysis were: *X^2^/df* = 5.47, CFI = 0.90, TLI = 0.82, RMSEA = 0.07, indicating that the scale has good reliability and validity.

### 2.6. Data processing

The raw data was obtained from an offline paper and pencil quiz. Mplus 8.3 and SPSS 26.0 were used for statistical analysis in this study. Firstly, Mplus 8.3 and SPSS 26.0 were used to test the reliability and validity of the questionnaires used in this study; secondly, common method bias tests were used to verify whether there were common method bias problems with the data, and the data from this study were analyzed for normality and tested for homogeneity between the experimental and control groups at pre-test using independent samples *t*-tests. If the data did not obey normality, the Whitney *U*-test was used; afterward, the data were subjected to repeated measures ANOVA and one-way ANOVA to test for differences before and after the experiment, within and between groups; Pearson correlation analysis was used to test for correlations between the variables and to verify whether they qualified for the mediation effect test; and finally, the Process plugin developed by Hayes (2013) was used to conduct The bootstrap method (5000 sample replicates) was used to estimate 95% confidence intervals for significance testing of mediation effects. Direct or indirect effects were considered significant when the confidence interval did not include zero.

## 3. Results

### 3.1. Homogeneity tests for demographic variables

Before the exercise intervention experiment began, the two classes selected were set up as the experimental and control groups, and independent samples *t*-tests were conducted on the demographic variables as well as the amount of physical activity between the experimental and control groups of middle school students to ensure that there were no differences between the two groups in terms of gender, age, height and weight and amount of physical activity. The results showed that (Table 2) the experimental and control groups were not significantly different in any of the demographic variables and were homogeneous.

**TABLE 2:** Homogeneity tests for demographic variables.

Variables	*p*
Gender	0.13
Age	0.72
Height (cm)	0.24
Body weight (kg)	0.36
Amount of physical activity	0.15

### 3.2. Data normality test for each variable before the experiment

Normality testing of pre-test data using the K-S test (Kolmogorov-Smirnov test). The results showed that the pre experimental middle school students’ interpersonal relationships, self-identity and social adjustment all obeyed a normal distribution. Therefore, an independent samples *t*-test was used to test the experimental variables’ homogeneity before the experiment started.

### 3.3. Common method bias test

All three variables (interpersonal relationships, self-identity, and social adjustment) were measured in this study using the subjects’ self-reports, which may be subject to common method bias. Therefore, the Harman one-way test was used for testing. The results showed that the common method bias factor explained 15.59% (less than the critical value of 40%), indicating no serious common method bias in this study.

### 3.4. Homogeneity test for each variable in different groups before the experiment

For the accuracy of the experimental results, it was necessary to check whether the subjects in the experimental and control groups differed in their initial levels on the three variables before the exercise intervention experiment began. Therefore, an independent samples *t*-test was conducted for the experimental and control groups. The results showed (Table 3) that there were no statistically significant differences in the levels of interpersonal relationships, self-identity and social adjustment between the subjects in the control and experimental groups before the start of the exercise intervention experiment (*p* > 0.05). The results indicate that the levels of each variable in the experimental and control groups before the exercise intervention experiment are homogeneous and can be analyzed further.

**TABLE 3:** Independent sample *t*-test for each variable for pre-experimental middle school students.

Variables	Group	M	SD	F	*p*	Cohen’s d
Interpersonal relationships	Experimental group	121.67	11.08	11.17	0.35	0.20
	Control group	118.71	17.62			
Self-identity	Experimental group	55.52	7.26	0.31	0.19	0.29
	Control group	53.27	8.42			
Social adjustment	Experimental group	166.14	10.23	0.27	0.42	0.02
	Control group	164.27	11.27			

### 3.5. The effects of basketball on interpersonal relationships, self-identity, and social adjustment of middle school students

The repeated measures ANOVA (Table 4) showed that in the time factor (pre-test and post-test), the interpersonal relationships (*F* = 80.93, *p* < 0.01, η*^2^p* = 0.49), self-identity (*F* = 188.96, *p* < 0.01, η*^2^p* = 0.69) and social adjustment (*F* = 190.66, *p* < 0.01, η*^2^p* = 0.69) levels of the middle school students in the experimental and control groups levels all showed significant changes over time trends. Among the group factors (experimental and control groups), there were substantial differences in the levels of interpersonal relationships (*F* = 11.24, *p* < 0.01, η*^2^p* = 0.12), self-identity (*F* = 20.57, *p* < 0.01, η*^2^p* = 0.20), and social adjustment (*F* = 25.89, *p* < 0.01, η*^2^p* = 0.23) between the experimental and control middle school students. In terms of the interaction term between time and group, there were statistically significant differences in interpersonal relationships (*F* = 12.70, *p* < 0.01, η*^2^p* = 0.13), self-identity (*F* = 16.12, *p* < 0.01, η*^2^p* = 0.16), and social adjustment (*F* = 67.24, *p* < 0.01, η*^2^p* = 0.44), indicating that the experimental group and control groups’ middle school students’ variable levels fluctuated significantly inconsistently with the time trend.

**TABLE 4:** Analysis of variance for the effect of basketball on each variable for middle school students.

Variables	Source of variation	Class III sum of squares	df	Mean square	F	*p*	η *2p*
Interpersonal relationships	Time	6920.46	1	6920.46	80.93	<0.01	0.49
	Error	7268.24	85	85.51			
	Group	2748.78	1	2748.78	11.24	<0.01	0.12
	Error	20794.91	85	244.65			
	Time x Group	1085.52	1	1085.52	12.70	<0.01	0.13
Self-identity	Time	5012.01	1	5012.01	188.96	<0.01	0.69
	Error	2254.60	85	26.53			
	Group	1265.84	1	1265.84	20.57	<0.01	0.20
	Error	1265.84	1	1265.84			
	Time × Group	428.51	1	428.51	16.12	<0.01	0.16
Social adjustment	Time	8458.03	1	8458.03	190.66	<0.01	0.69
	Error	3770.81	85	44.36			
	Group	4486.66	1	4486.66	25.89	<0.01	0.23
	Error	14731.84	85	173.32			
	Time × Group	2982.86	1	2982.86	67.24	<0.01	0.44

**FIGURE 2:**
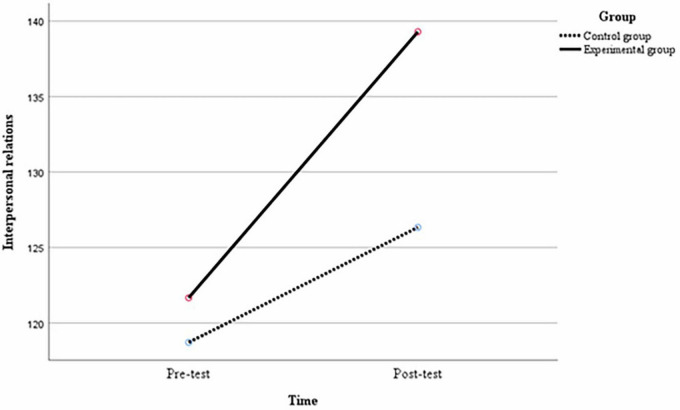
Changes in the level of interpersonal relationship between different groups before and after the experiment.

**FIGURE 3:**
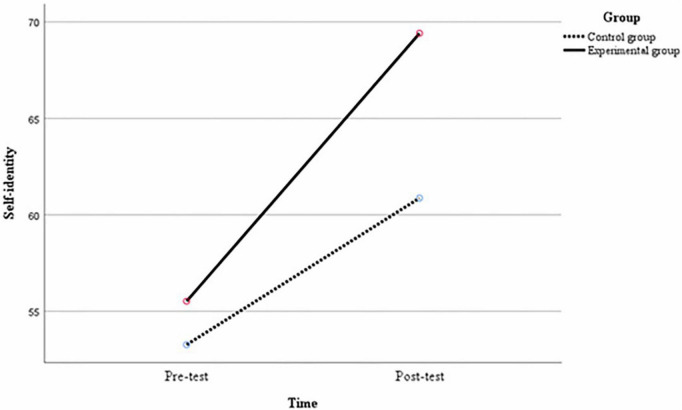
Changes in the level of self-identity of different groups before and after the experiment.

**FIGURE 4:**
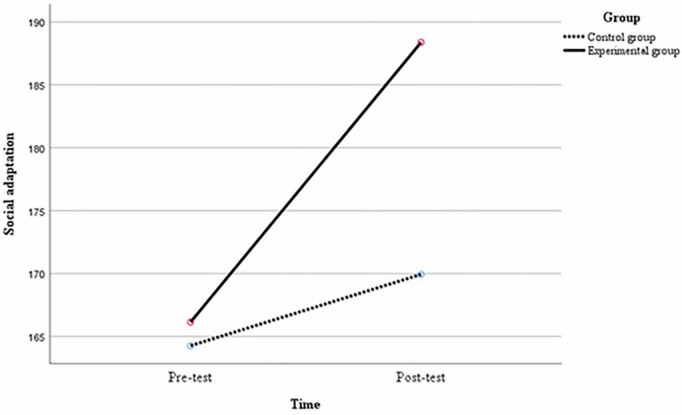
Changes in the level of social adjustment in different groups before and after the experiment.

Multiple comparisons were made between the variables to compare the differences in each variable between groups at the same time and between middle school students at different times under the same group conditions. At the pre-test, there were no significant differences between the experimental and control groups of middle school students in terms of the mean difference in interpersonal relationships of 2.96, *p* = 0.29; the mean difference in self-identity of 2.26, *p* = 0.12; and the mean difference in the social adjustment of 1.88, *p* = 0.40. At post-test, there were significant differences between the experimental and control groups of middle school students in the mean difference in interpersonal relationships of 12.95, *p* < 0.01; the mean difference in self-identity of 8.54, *p* < 0.01; and the mean difference in the social adjustment of 18.45, *p* < 0.01. As can be seen from Table 7, in the experimental group, there were significant differences in the mean difference between the post-test and the pre-test for interpersonal relationships of 17.62, *p* < 0.01; the mean difference for self-identity of 13.88, *p* < 0.01; and the mean difference for social adjustment of 22.24, *p* < 0.01 for middle school students. In the control group, the difference in interpersonal relationships between the post-test and pre-test of middle school students was 7.62, *p* < 0.01; the difference in mean self-identity was 7.60, *p* < 0.01; and the difference in mean social adjustment was 5.67, *p* = 0.01, again with significant differences. As shown in Figures 2–4, after the 12-week experimental cycle, the interpersonal relationships, self-identity and social adjustment levels of the experimental and control groups improved significantly, and the experimental group improved significantly better than the control group in all variables.

### 3.6. Correlation analysis for each variable

Using correlation analysis to examine the interpersonal relationships, self-identity and social adjustment of middle school students (Table 5), the results showed that there were significant correlations between interpersonal relationships, self-identity and social adjustment of middle school students (*p* < 0.01). Further analysis can therefore be carried out.

**TABLE 5:** Correlation test for each variable.

Variables	1	2	3	4
1 Basketball interventions				
2 Interpersonal relationships	0.53**			
3 Self-identity	0.65**	0.72**		
4 Social adjustment	0.68**	0.71**	0.80**	

**p < 0.01.

### 3.7. Multicollinearity test

The correlation analysis showed that there were significant correlations between all the variables. Therefore, the issue of colinearity may arise, thus affecting the accuracy of the regression analysis results. Therefore, this study standardized the predictor variables in each equation to overcome the covariance problem and performed a multiple covariance test. The results show that the tolerance for all predictor variables is 0.32–0.43 > 0.1, and the VIF (Variance Inflating Factor) is 2.31–3.15 < 5. Therefore, the data in this study do not suffer from severe colinearity problems and can be tested for mediating and chain mediating effects.

### 3.8. A model test of the effect of a basketball intervention on social adjustment of middle school students

**FIGURE 5:**
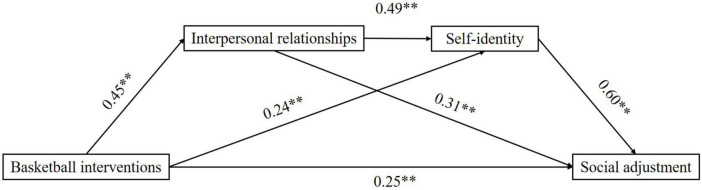
Path coefficient diagram. ^**^p < 0.01.

This study used the spss plug-in process developed by Hayes (2013) to construct and test the chain mediator model. Model 6 was selected, with the group factor as the independent variable, social adjustment as the dependent variable, and interpersonal relationships and self-identity as the chain mediator variables and the results of the path coefficients are shown in Figure 5.

The test results for mediating effects using Bootstrap sampling (Table 6) showed that the total effect of the basketball intervention on social adjustment was 0.66. Regression analysis showed that the basketball intervention had a positive impact on interpersonal relationships, self-identity and social adjustment (β = 0.45, t = 5.83, *p* < 0.01; β = 0.24, *t* = 33.84, *p* < 0.01; β = 0.25, *t* = 3.13, *p* < 0.01).

**TABLE 6:** Regression analysis of basketball interventions and chain mediation models of inter-personal relationships, self-identity, and social adjustment (post-test/standardized).

Variables	Interpersonal relationships		Self-identity		Social adjustment		Total effect	
	β	t	β	t	β	t	β	t
Basketball interventions	0.45	5.83	0.24	3.84	0.25	3.13	0.66	8.00
Interpersonal relationships			0.49	6.35	0.31	2.82		
Self-identity					0.60	4.68		
Social adjustment								
Control variables								
Age	0.03	0.29	−0.11	−1.53	−0.02	−0.24		
Gender	−0.32	−2.26	−0.01	−0.09	−0.06	−0.47		
*R* ^2^	0.32		0.62		0.72		0.48	
*F*	13.20**		33.88**		40.91**		25.71**	

**p < 0.01.

**TABLE 7:** Chain mediated model effect tests for basketball sport interventions and interpersonal relationships, self-identity and social adjustment.

Effect type	Effect size	BootSE	Bootstrap 95% CI		Percentage of relative effect
			Lower limit	Upper limit	
Total effect	0.66	0.08	0.50	0.83	100%
Direct effects	0.25	0.08	0.09	0.41	37.9%
Indirect effects 1	0.14	0.05	0.04	0.25	21.2%
Indirect effect 2	0.15	0.06	0.05	0.27	22.7%
Indirect effects 3	0.13	0.05	0.05	0.23	19.7%
Total indirect effect	0.41	0.08	0.27	0.57	62.1%

Indirect effects 1 = Basketball interventions➡ Interpersonal relationships➡ Social adjustment. Indirect effects 2 = Basketball interventions➡ Self-identity➡ Social adjustment. Indirect effects 3 = Basketball interventions➡ Interpersonal relationships➡ Self-identity➡ Social adjustment.

Chain mediated model effects tests (Table 7) showed a significant direct effect of the basketball intervention on the impact of social adjustment with an effect size of 0.25 (*Bootstraps CI* [0.09–0.41]), the relative effect of 37.9%. Indirect effect 1 (basketball intervention → interpersonal relationships → social adjustment) was significant, the effect size of 0.14 (*Bootstrap CI* [0.04–0.25]), and the relative effect accounted for 21.2%. Indirect effect 2 (basketball intervention → self-identity → social adjustment) was significant, effect size of 0.15 (*Bootstrap CI* [0.05–0.27]). The relative effect accounted for 22.7%. Indirect effect 3 (basketball intervention → interpersonal relationships → self-identity → social adjustment) was a significant effect size of 0.13 (*Bootstrap CI* [0.05–0.23]). The relative effect accounted for 19.7%. The total effect size for all indirect effects was 0.41 (*Bootstrap CI* [0.27–0.57]), and the relative effect accounted for 62.1%. The chain mediating role of interpersonal relationships and self-identity in the positive effect of basketball intervention on social adjustment holds.

## 4. Discussion

This study found that middle school students in both the experimental and control groups produced improved levels of interpersonal relationships, self-identity and social adjustment at the end of the 12-week experiment. However, the extent of improvement in all variables was more significant for the middle school students in the experimental group than for the control group. At the same time, interpersonal relationships and self-identity mediated the pathway of basketball intervention affecting middle school students’ social adjustment, respectively, and the chain mediation effect was held.

In conclusion, it can be reasonably inferred from the current research findings on the intensity, duration, frequency and periodicity of physical activity and the results of this experiment that the significant increase in each of the variables studied in both the experimental and control groups was due to the positive psychological effects of physical activity. The reason why the control group showed a significantly smaller increase in each variable than the experimental group may be due to the fact that the exercises in the regular physical education classes were of medium intensity, but they were conducted in a single method and lacked a coordinated and cooperative group exercise. The experimental group, on the other hand, performed moderate-to-high intensity physical exercise and a multi-team collaborative basketball-specific exercise intervention that may be more suitable for the physical and mental characteristics of junior high school students. As a result, the experimental group of junior high school students showed significantly higher levels of improvement in all variables than the control group.

### 4.1. Effects of basketball intervention on social adjustment of middle school students

The study showed that after 12 weeks of basketball intervention, middle school students significantly increased their level of social adjustment. Cross-stressor-adaptation theory suggests that physical exercise is a source of stress for the individual. Still, over time and with continued effort on the part of the individual, the stress caused by physical exercise will eventually be adapted to the individual (Moake and Patel, 2021). When individuals adapt to this stress, they can develop a more resilient mental state that can help them adapt to and overcome other sources and types of stress in their lives, thus having a beneficial effect on their social adjustment. In addition, according to social adjustment theory, physical exercise as a positive social interaction activity can provide a platform full of interpersonal interaction and social interaction for young people (Bristol, 1915). In addition, according to social adjustment theory, physical activity as a positive social interaction activity can provide a platform full of interpersonal interaction and social interaction for young people. For Chinese middle school students, physical education classes are more practical than other courses, in which young people can exchange emotions with their peers, share joys, build a broader network of relationships and improve their social adjustment skills during exercise practice. At the same time, physical exercise as a challenging practical activity is often accompanied by task challenge, difficulty conquest, self-transcendence and goal achievement so that individuals can continuously learn and cope with challenges in physical exercise, achieve the balance between themselves and the environment through assimilation and conformity, and enhance their ability to deal with and adapt to the situation (Piaget, 1964). The high level of cooperative learning and competition practice also played an essential role in the social adjustment development of middle school students during the 12-week exercise intervention experiment. Basketball not only provides a medium for students to interact and communicate with each other, strengthening their ability to communicate with others (Pan et al., 2016), but also promotes the development of their personalities as well as their self-confidence and sense of teamwork, civilized self-discipline and social adaptation through the mental exercise of working hard, accepting success or failure, praise or criticism, etc. According to Bornstein et al. (2010), in addition to strengthening the body, physical exercise can also stimulate a sense of teamwork, develop interpersonal communication skills, and enrich problem solving skills and techniques, thereby improving overall social and coping and interpersonal skills and reducing externalized problem behavior. Combined with the findings of this study, we suggest that encouraging adolescents to participate in periodic moderate intensity group physical exercise can enhance their social adjustment skills.

### 4.2. Effects of basketball intervention on interpersonal relationships of middle school students

The study showed that after 12 weeks of basketball intervention, middle school students significantly increased their interpersonal relationships level. It has been suggested that physical exercise provides a platform for individuals to interact with each other, enhances interpersonal communication and promotes higher levels of interpersonal relationships (Li and Qiu, 2018). This view also fits in with the social adaptation theory proposed by Bristol (1915). Joo (2018) found that college students who performed moderate and vigorous physical exercise had the best interpersonal relationships level; this is consistent with the findings of this paper, suggesting that the results also apply to middle school students. The reason basketball can improve the interpersonal skills of middle school students is that basketball, as a team sport, relies on frequent and effective communication between teachers and students and between students and students to achieve exemplary teaching results and game performance. This process is conducive to forming good interpersonal skills among basketball participants (Kelly, 2013). In addition, developing self-control may play a key role in basketball’s ability to improve interpersonal relationships among middle school students. As basketball is also a complex sport skill sport with variable technical and tactical characteristics, a high level of individual self-control is often required in the game to cope with changing on court situations, apply flexible strategies and adapt to the changing demands of the task. It has been shown that long term physical exercise is effective in improving and promoting self-control in individuals (Schoendube et al., 2017); individuals with a high level of self-control are less likely to experience interpersonal conflict and interpersonal distress (Tangney et al., 2004), there is a positive correlation between the level of interpersonal skills and the level of interpersonal relationships. Therefore, the 12-week basketball intervention effectively improved the interpersonal skills of middle school students.

### 4.3. Effects of basketball intervention on middle school students’ self-identity

The study’s results showed that after 12 weeks of basketball intervention, middle school students showed a significant increase in their level of self-identity. Based on a review of domestic and international research on self-identity, it can be concluded that self-identity refers to the individual’s recognition and acceptance of the reflected self and the relationship between the self and society, from which it can be seen that self-identity includes self-perception and self-evaluation (Chen, 2011). Therefore, the findings that the basketball intervention increased middle school students’ self-identity can be explained in terms of changes in individual self-perceptions. It has been shown that there is a positive relationship between physical exercise and an individual’s self-identity (Batsiou et al., 2020); that is, within a specific range, the more physical exercise there is, the higher the level of self-identity, in line with the findings of this paper. In the process of basketball, individuals improve their skills through their efforts, work closely with their teammates to beat their opponents in games, and respond to the changing technical and tactical demands on the court, from being overwhelmed at first to being comfortable with the game (Anderson et al., 2007). This virtuous circle can help middle school students build self-confidence, reduce their sense of inferiority, help them form positive and healthy values and worldviews, perceive their strengths more efficiently, and ultimately significantly improve their self-identity.

### 4.4. The chain mediating role of interpersonal relationships and self-identity

Analysis of the chain mediating effect revealed that interpersonal relationships and self-identity play a chain mediating role in the theoretical model of this study in which the basketball intervention affects the social adjustment of middle school students. Reviewing previous research, we found that peer relationships are essential to middle school students’ interpersonal networks (Fuligni et al., 2001). Therefore, peer acceptance can significantly influence the interpersonal relationships among middle school students. Research has found that being accepted and liked by peers in a group strengthens individuals’ positive perceptions when perceiving themselves and their surroundings and demonstrates higher levels of interpersonal relationships by dealing with interpersonal issues in a positive mindset and manner and integrating better into the group (Will et al., 2018; Lindsey and Berks, 2019). The 12-week basketball intervention gave middle school students more opportunities to interact with their peers, complete practice tasks set by teachers cooperatively, and continuously strengthen their bonds with each other during games, forming good interpersonal relationships of mutual help, encouragement and dependence. Positive interpersonal relationships provide an excellent socialization environment for middle school students, helping them to acquire better social skills and the social cognitive skills necessary for interaction (Will et al., 2018; Lindsey and Berks, 2019). In this process, individuals can demonstrate excellent emotional stability and a greater willingness to explore new things and interact socially (Santos et al., 2014), leading to better social adjustment skills. From the study results, it is clear that an increase in the level of interpersonal relationships also contributed to an improvement in the individual’s self-identity. According to the stress-buffering Hypothesis (Cassel, 1976), Individuals with high levels of interpersonal relationships can release the pain and negative consequences of general stress, and self-identity, as an essential part of the stress buffer, has the same effect as interpersonal relationships (Schwartz et al., 2011). It may indicate that interpersonal relationships have an enhancing impact on an individual’s self-identity. The types of support generated by high levels of interpersonal relationships can also lead individuals to believe that they can cope or adjust to life’s emergencies (Cohen, 2004), leading to a higher level of self-identity. The results of this study also suggest that an increase in self-identity also helped improve individuals’ social adjustment levels. It was found that an individual’s self-identity predicted emotional adjustment indicators closely related to social adjustments, such as anxiety, depression and subjective wellbeing (Cramer, 1995), and that individuals with high levels of self-identity tend to have a positive self-valence, meet challenges with a positive attitude, and are less likely to lose hope and lose themselves, thus increasing their level of social adjustment (Monica Pellerone et al., 2015). From the perspective of the risk and protective factor model (Rolf et al., 1990), various risk and protective factors exist simultaneously in an individual’s family and other social environments. Self-identity as a type of protective factor and risk factors jointly determine the development of an individual’s social adjustment through mutual influence and interaction. In summary, interpersonal relationships and self-identity play a chain mediating role in the pathways by which basketball interventions affect individuals’ social adjustment.

## 5. Conclusion

This study found that both the basketball intervention and regular PE classes improved middle school students’ interpersonal relationships, self-identity and social adjustment levels. Still, the basketball intervention was significantly more effective than regular PE classes. Furthermore, the basketball intervention was able to predict social adjustment through interpersonal relationships and self-identity, respectively, i.e., interpersonal relationships and self-identity acted as a chain mediator in the pathways through which the basketball intervention influenced social adjustment.

## 6. Limitations and perspectives

In previous studies, scholars have mostly used cross-sectional surveys to explore the relationship between physical activity and interpersonal relationships, self-identity and social adjustment. This paper, however, may be the first time that a natural experiment has been conducted using a basketball sport intervention and validates the findings of previous studies. However, the conclusions of this paper are derived from the results of statistical analysis, and further research is needed to determine whether they have strong external validity.

## Data availability statement

The original contributions presented in this study are included in the article/Supplementary material, further inquiries can be directed to the corresponding author.

## Ethics statement

The studies involving human participants were reviewed and approved by Ethical Committee of Yangzhou University Medical College. Written informed consent to participate in this study was provided by the participants’ legal guardian/next of kin.

## Author contributions

All authors listed have made a substantial, direct, and intellectual contribution to the work, and approved it for publication.
