# Supplementary material for: The impact of basketball on the social adjustment of Chinese middle school students: the chain mediating role of interpersonal relationships and self-identity
Source: Front Psychol. 2023 Jun 28;14:1205760. doi: 10.3389/fpsyg.2023.1205760 (PMC10338091; doi:10.3389/fpsyg.2023.1205760)
Supplement: Supplementary file 1 [file Data_Sheet_1.ZIP › Code book.docx]

Code book

**Data file name: Raw data**

**Code Interpretation：**

**Line9- Line12:**

**Physical Activity Rating Scale（PARS—3）**

**T1:** How is the intensity of your physical exercise?

① Light exercise (e.g. walking, doing radio gymnastics, playing gateball, etc.)

② Less intense exercise (e.g. volleyball, table tennis, jogging, tai chi, etc. for recreational purposes)

③ Medium-intensity, more intense and sustained exercise (e.g. cycling, running, table tennis, etc.)

④ High-intensity, but not sustained exercise with shortness of breath and sweating (e.g. playing badminton, basketball, tennis, football, etc.)

⑤ High-intensity, long-lasting exercise with heavy breathing and sweating (e.g. running, sets of aerobics exercises, swimming, etc.)

**T2:** How many minutes at a time do you perform the above-mentioned intense physical activities?

① Less than 10 minutes

② 11 to 20 minutes

③ 21 to 30 minutes

④ 31 to 59 minutes

⑤ 60 minutes or more

**T3:** How many times a month do you do the above-mentioned physical activities?

① Less than once a month

② 3 to 5 times a week

③ 2 to 3 times a month

④ About once a day

⑤ 1 to 2 times a week

**T4:** What kind of physical exercise do you like?

① Walking, running

② Travel, excursions

③ Sports dance

④ Ball games

⑤ Rope skipping

⑥ Taijiquan, health and fitness

⑦ Exercise machine activities

⑧ Swimming

⑨ Others ___________________________

**Line13- Line72:**

**Mental Health Rating Scale for Secondary School Students**

| Name：_______________Gender：______Age：_____School：_________________________  Class：___________ | |
| --- | --- |
| 1. I don't like to participate in extra-curricular activities at school. | None←……→Serious |
| 2. I am in a good mood and in a bad mood. | 1 2 3 4 5 |
| 3. I have to check my homework over and over again. | 1 2 3 4 5 |
| 4. I feel that people are not friendly to me and do not like me. | 1 2 3 4 5 |
| 5. I feel miserable. | 1 2 3 4 5 |
| 6. I feel nervous or get nervous easily. | 1 2 3 4 5 |
| 7. My motivation to study is high and low. | 1 2 3 4 5 |
| 8. I feel uncomfortable with my current school life. | 1 2 3 4 5 |
| 9. I am not used to the current social atmosphere. | 1 2 3 4 5 |
| 10. I have to do things very slowly to make sure I get it right. | 1 2 3 4 5 |
| 11. I always think differently from others. | 1 2 3 4 5 |
| 12. Always worried about whether my clothes are neat or not. | 1 2 3 4 5 |
| 13. Cries easily. | 1 2 3 4 5 |
| 14. I feel hopeless about my future. | 1 2 3 4 5 |
| 15. I feel fidgety and restless. | 1 2 3 4 5 |
| 16. Blame myself a lot. | 1 2 3 4 5 |
| 17. I feel uneasy when people look at me or talk about me. | 1 2 3 4 5 |
| 18. I feel that people do not understand me. | 1 2 3 4 5 |
| 19. I often lose my temper and try to control it but I can't. | 1 2 3 4 5 |
| 20. Feel that others are trying to take advantage of me. | 1 2 3 4 5 |
| 21. Yells or drops things. | 1 2 3 4 5 |
| 22. Always thinking about unnecessary things. | 1 2 3 4 5 |
| 23. Have to wash my hands or count over and over again. | 1 2 3 4 5 |
| 24. I always feel like someone is talking behind my back. | 1 2 3 4 5 |
| 25. Argues and argues with people from time to time. | 1 2 3 4 5 |
| 26. I feel that I cannot trust most people. | 1 2 3 4 5 |
| 27. My enthusiasm for doing homework fluctuates. | 1 2 3 4 5 |
| 28. I feel sad when my classmates do better than me in exams. | 1 2 3 4 5 |
| 29. I don't adapt to the teacher's teaching methods. | 1 2 3 4 5 |
| 30. The teacher is unfair to me. | 1 2 3 4 5 |
| 31. I feel a heavy burden of study. | 1 2 3 4 5 |
| 32. I am cold and hot to my classmates. | 1 2 3 4 5 |
| 33. In class, I am always worried that the teacher will ask me questions. | 1 2 3 4 5 |
| 34. I suddenly feel scared for no reason. | 1 2 3 4 5 |
| 35. I am sometimes close to and sometimes distant from my teachers. | 1 2 3 4 5 |
| 36. I feel nervous when I hear that I have an exam. | 1 2 3 4 5 |
| 37. I feel uncomfortable when other students dress better than me and have money. | 1 2 3 4 5 |
| 38. I hate doing homework. | 1 2 3 4 5 |
| 39. My home environment interferes with my studies. | 1 2 3 4 5 |
| 40. I hate going to school. | 1 2 3 4 5 |
| 41. I don't like the atmosphere in my class. | 1 2 3 4 5 |
| 42. My parents are not fair to me. | 1 2 3 4 5 |
| 43. I feel irritable. | 1 2 3 4 5 |
| 44. I am often lethargic and unmotivated. | 1 2 3 4 5 |
| 45. My feelings are easily hurt by others. | 1 2 3 4 5 |
| 46. I feel unsure of myself. | 1 2 3 4 5 |
| 47. People do not judge my performance properly. | 1 2 3 4 5 |
| 48. I know it is useless to worry, but I am always afraid that I will not do well on the test. | 1 2 3 4 5 |
| 49. I always feel that others are against me. | 1 2 3 4 5 |
| 50. I get easily upset and worried. | 1 2 3 4 5 |
| 51. I feel shy and uncomfortable around people of the opposite sex. | 1 2 3 4 5 |
| 52. I have an urge to hurt or hit others. | 1 2 3 4 5 |
| 53. I am sometimes affectionate and sometimes cold to my parents. | 1 2 3 4 5 |
| 54. I do not take kindly to classmates who are better than me. | 1 2 3 4 5 |
| 55. I hate exams. | 1 2 3 4 5 |
| 56. I always feel that something is wrong in my heart. | 1 2 3 4 5 |
| 57. I often have suicidal thoughts. | 1 2 3 4 5 |
| 58. I have the urge to break things. | 1 2 3 4 5 |
| 59. Demands perfection from others. | 1 2 3 4 5 |
| 60. My classmates have higher grades than me in exams, but they are not better than me. | 1 2 3 4 5 |

**Line73- Line102:**

**Interpersonal Relationship Scale**

|  | not meets←……→meets |
| --- | --- |
| 1. I feel nervous when talking to a person of the opposite sex. | 1____2____3____4____5____ |
| 2. It is impossible for me to find a classmate of the same sex to talk about my daily problems. | 1____2____3____4____5____ |
| 3. I think my parents interfere too much in my affairs. | 1____2____3____4____5____ |
| 4. I think teachers never really know me. | 1____2____3____4____5____ |
| 5. I don't like working with students of the opposite sex during group discussions. | 1____2____3____4____5____ |
| 6. I don't have any friends of the same sex. | 1____2____3____4____5____ |
| 7. My parents don't really know me. | 1____2____3____4____5____ |
| 8. My teachers ignore me even when I do well. | 1____2____3____4____5____ |
| 9. I look down on the opposite sex. | 1____2____3____4____5____ |
| 10. I am not willing to help a classmate of the opposite sex in any situation. | 1____2____3____4____5____ |
| 11. Some of my friends of the same sex do not stand by me in any difficult situation. | 1____2____3____4____5____ |
| 12. I rarely communicate with my parents. | 1____2____3____4____5____ |
| 13. I secretly think about the opposite sex. | 1____2____3____4____5____ |
| 14. I am often talked about and fooled by people of the same sex. | 1____2____3____4____5____ |
| 15. It is impossible for me to be friends with the opposite sex. | 1____2____3____4____5____ |
| 16. I do not work well with my same-sex classmates in a group. | 1____2____3____4____5____ |
| 17. I do not know how to get along better with people of the opposite sex. | 1____2____3____4____5____ |
| 18. When strangers ask me for help, I always try to say no. | 1____2____3____4____5____ |
| 19. Sometimes I feel that teachers are deliberately trying to get me into trouble. | 1____2____3____4____5____ |
| 20. I am reluctant to accept help from strangers in public. | 1____2____3____4____5____ |
| 21. I am looked down upon by the opposite sex. | 1____2____3____4____5____ |
| 22. I prefer to go out with my classmates rather than with my parents. | 1____2____3____4____5____ |
| 23. I often feel sad because I have been hurt. | 1____2____3____4____5____ |
| 24. I am often talked about and made a fool of by others. | 1____2____3____4____5____ |
| 25. Teachers treat me with contempt. | 1____2____3____4____5____ |
| 26. Does not get on well with people of the same sex. | 1____2____3____4____5____ |
| 27. I am rejected and treated with indifference by people of the same sex. | 1____2____3____4____5____ |
| 28. When I get lost, I prefer to read a map myself rather than ask a stranger for directions. | 1____2____3____4____5____ |
| 29. I feel uncomfortable with people of the opposite sex. | 1____2____3____4____5____ |
| 30. Often hurt my friends of the same sex. | 1____2____3____4____5____ |

**Line103- Line121:**

**Self-Identity Scale**

|  | Completely inapplicable←……→Very suitable |
| --- | --- |
| 1. I don't know what kind of person I am | 1____2____3____4____ |
| 2. People always change their opinion of me | 1____2____3____4____ |
| 3. I know how I should live my life | 1____2____3____4____ |
| 4. I am not sure if something is morally right | 1____2____3____4____ |
| 5. most people agree on what kind of person I am | 1____2____3____4____ |
| 6. I feel that the way I live suits me | 1____2____3____4____ |
| 7. My worth is recognised by others | 1____2____3____4____ |
| 8. I feel freer to be who I really am when I am not surrounded by people I know | 1____2____3____4____ |
| 9. I feel that the things I do in life are not really worthwhile | 1____2____3____4____ |
| 10. I feel well adjusted to the community in which I live | 1____2____3____4____ |
| 11. I am proud of the person I have become | 1____2____3____4____ |
| 12. People's perceptions of me are very different from my perceptions of myself | 1____2____3____4____ |
| 13. I feel ignored | 1____2____3____4____ |
| 14. People don't seem to accept me | 1____2____3____4____ |
| 15. I have changed the way I want to get something out of life | 1____2____3____4____ |
| 16. I am not quite sure what people think of me | 1____2____3____4____ |
| 17. my feelings about myself have changed | 1____2____3____4____ |
| 18. I feel that I act or do things for utilitarian reasons | 1____2____3____4____ |
| 19. I am proud to be part of the society I live in | 1____2____3____4____ |

**Line122- Line164:**

**Adolescent Social Adjustment Scale**

|  | Not at all←……→Fully compliant |
| --- | --- |
| 1. I try to find good ways to learn when it comes to studying. | 1 2 3 4 5 |
| 2. I know how to make more friends. | 1 2 3 4 5 |
| 3. I want to develop myself through class activities. | 1 2 3 4 5 |
| 4. I have never been bored with my studies. | 1 2 3 4 5 |
| 5. When I have different opinions from my classmates, I can find ways to prevent conflicts from arising. | 1 2 3 4 5 |
| 6. The class I am in now is very united. | 1 2 3 4 5 |
| 7. I am always close to everyone. | 1 2 3 4 5 |
| 8. I think it is normal for classmates to have different views on some issues. | 1 2 3 4 5 |
| 9. I think that I am a lively and cheerful person. | 1 2 3 4 5 |
| 10. I usually finish my homework on time. | 1 2 3 4 5 |
| 11. My classmates welcome me to play with them. | 1 2 3 4 5 |
| 12. I am willing to take part in some voluntary work. | 1 2 3 4 5 |
| 13. I wash my own clothes. | 1 2 3 4 5 |
| 14. I can do what my teachers or parents say. | 1 2 3 4 5 |
| 15. I feel that I live in a fairer society. | 1 2 3 4 5 |
| 16. I have never told a lie. | 1 2 3 4 5 |
| 17. I understand people who have different views and habits from mine. | 1 2 3 4 5 |
| 18. I am an optimistic person. | 1 2 3 4 5 |
| 19. I always complete my homework independently and do not copy my classmates'. | 1 2 3 4 5 |
| 20. I think that I have more friends. | 1 2 3 4 5 |
| 21. I think it is meaningful to take part in the activities organised by the class. | 1 2 3 4 5 |
| 22. I cook my own meals when my parents work. | 1 2 3 4 5 |
| 23. I hate studying. | 1 2 3 4 5 |
| 24. When my classmates argue about some issues, I can often help them to reconcile their arguments. | 1 2 3 4 5 |
| 25. I am happy with my living environment. | 1 2 3 4 5 |
| 26. I feel that there are people I should not get close to. | 1 2 3 4 5 |
| 27. It is normal for different people to do the same thing in different ways. | 1 2 3 4 5 |
| 28. I think I have a sense of humour. | 1 2 3 4 5 |
| 29. I study every subject carefully. | 1 2 3 4 5 |
| 30. I feel that most of my friends trust me. | 1 2 3 4 5 |
| 31. I often want to win for the group. | 1 2 3 4 5 |
| 32. At home, I often help my parents with household chores. | 1 2 3 4 5 |
| 33. If I don't think what my teachers or parents say makes sense, I won't do what they say. | 1 2 3 4 5 |
| 34. I help my classmates to make up with each other when they have conflicts. | 1 2 3 4 5 |
| 35. I used to tell lies to avoid being blamed by my teachers or parents. | 1 2 3 4 5 |
| 36. I think it is normal for classmates to argue with each other about issues all the time. | 1 2 3 4 5 |
| 37. I like to talk to people. | 1 2 3 4 5 |
| 38. Sometimes my teacher doesn't set homework, but I still take the initiative to study when I get home. | 1 2 3 4 5 |
| 39. I maintain good relationships with my classmates. | 1 2 3 4 5 |
| 40. I am happy to take part in most group activities. | 1 2 3 4 5 |
| 41. I can clean and tidy up my room. | 1 2 3 4 5 |
| 42. I study very hard. | 1 2 3 4 5 |
| 43. I am satisfied with the school I am attending. | 1 2 3 4 5 |

**Line166= Standardized interpersonal scale score**

**Line167= Standardized Social Adjustment scale score**

**Line168= Standardized self-identity scale scores**

**Line169=** **Standardized group**
