# Supplementary material for: The impact of basketball on the social adjustment of Chinese middle school students: the chain mediating role of interpersonal relationships and self-identity
Source: Front Psychol. 2023 Jun 28;14:1205760. doi: 10.3389/fpsyg.2023.1205760 (PMC10338091; doi:10.3389/fpsyg.2023.1205760)
Supplement: Supplementary file 1 [file Data_Sheet_1.ZIP › Informed Consent Form.docx]

**Informed Consent Form**

**Research Background**

Your child is about to take part in a study that will be conducted to demonstrate that physical activity promotes social adjustment in junior high school students and it will last for 12 weeks. Your child is invited to participate in this experiment as he/she is eligible to be a participant.

This informed consent form will provide information to help you decide whether to participate in the study. Your child's participation in this study is voluntary. This study has been reviewed by our Institutional Ethics Review Board. If you agree to your child's participation in this study, please read the following instructions in detail:

**Objectives of the study**

Middle school is a critical period in the development of students' physical and mental health, which may bring about social maladjustment. Social adaptation theory and integrated adaptive development theory suggest that an individual's adaptive capacity develops through continuous learning and exploration in life practice. From that level, physical activity as a positive social interaction activity may be able to enhance the social adjustment ability of individuals. This study also takes this as a starting point to explore the effects of physical activity on secondary school students' social adjustment skills and the mechanisms involved, using an experimental approach of basketball intervention.

**Research process and methodology**

Before you are enrolled, a team member will administer a questionnaire to obtain general information about your child and your child may volunteer to participate in the study by signing an informed consent form. If your child volunteers, your child will be randomised and may be placed in either the control group or the intervention group. The control group will have regular classroom teaching and the intervention group will have a physical activity intervention based on the control group.

**Research risks and discomfort**

By participating in this experiment, your child may experience a degree of fatigue.

**Privacy issues**

If you decide to involve your child in this study, your child's participation and personal details will be kept strictly confidential and will not be disclosed to third parties unless you and your child have given us permission to do so. Therefore, in order to ensure the authenticity of the experiment, it is important that you and your child provide us with your true details. The research data will also be stored in the research centre and will only be accessible by the researchers. You and your child must also observe the principle of confidentiality with regard to information relating to this experiment and must not divulge it to third parties.

**Free withdrawal**

As a subject, your child will be kept informed of information and research progress related to this study and can voluntarily decide whether to continue to participate in the experiment.

If you have any questions about this study, you can contact the study leader, Mr. Hao Ran, at 15050980680.

**Informed Consent Signature:**

I have read this informed consent form carefully and the investigator has explained the purpose, content and risks of the experiment to me in detail and has answered my questions, I understand the experiment and I consent to my child's participation in the experiment.

Parent's signature:

Date: Month of year
